# Physiological MRI of microvascular architecture, neovascularization activity, and oxygen metabolism facilitate early recurrence detection in patients with IDH-mutant WHO grade 3 glioma

**DOI:** 10.1007/s00234-021-02740-9

**Published:** 2021-06-11

**Authors:** Andreas Stadlbauer, Gertraud Heinz, Stefan Oberndorfer, Max Zimmermann, Thomas M. Kinfe, Michael Buchfelder, Arnd Dörfler, Natalia Kremenevski, Franz Marhold

**Affiliations:** 1 Institute of Medical Radiology, University Clinic St. Pölten, Karl Landsteiner University of Health Sciences, Dunant Platz 1, A-3100, St. Pölten, Austria; 2 Department of Neurosurgery, Friedrich-Alexander University (FAU) Erlangen-Nürnberg, Erlangen, Germany; 3 Department of Neurology, University Clinic of St. Pölten, Karl Landsteiner University of Health Sciences, St. Pölten, Austria; 4 Department of Preclinical Imaging and Radiopharmacy, University of Tübingen, Tübingen, Germany; 5 Division of Functional Neurosurgery and Stereotaxy, Friedrich-Alexander University (FAU) Erlangen-Nürnberg, Erlangen, Germany; 6 Department of Neuroradiology, Friedrich-Alexander University (FAU) Erlangen-Nürnberg, Erlangen, Germany; 7 Department of Neurosurgery, University Clinic of St. Pölten, Karl Landsteiner University of Health Sciences, St. Pölten, Austria

**Keywords:** Humans, Glioma, Magnetic resonance imaging, Oxygen metabolism, Neoplasm recurrence, Treatment failure, Neovascularization

## Abstract

**Purpose:**

This study aimed to determine the diagnostic performance of physiological MRI biomarkers including microvascular perfusion and architecture, neovascularization activity, tissue oxygen metabolism, and tension for recurrence detection of IDH-mutant WHO grade 3 glioma.

**Methods:**

Sixty patients with IDH-mutant WHO grade 3 glioma who received overall 288 follow-up MRI examinations at 3 Tesla after standard treatment were retrospectively evaluated. A conventional MRI protocol was extended with a physiological MRI approach including vascular architecture mapping and quantitative blood-oxygen-level-dependent imaging which required 7 min extra data acquisition time. Custom-made MATLAB software was used for the calculation of MRI biomarker maps of microvascular perfusion and architecture, neovascularization activity, tissue oxygen metabolism, and tension. Statistical procedures included receiver operating characteristic analysis.

**Results:**

Overall, 34 patients showed recurrence of the WHO grade 3 glioma; of these, in 15 patients, recurrence was detected one follow-up examination (averaged 160 days) earlier by physiological MRI data than by conventional MRI. During this time period, the tumor volume increased significantly (*P* = 0.001) on average 7.4-fold from 1.5 to 11.1 cm^3^. Quantitative analysis of MRI biomarkers demonstrated microvascular but no macrovascular hyperperfusion in early recurrence. Neovascularization activity (AUC = 0.833), microvascular perfusion (0.682), and oxygen metabolism (0.661) showed higher diagnostic performance for early recurrence detection of WHO grade 3 glioma compared to conventional MRI including cerebral blood volume (0.649).

**Conclusion:**

This study demonstrated that the targeted assessment of microvascular features and tissue oxygen tension as an early sign of neovascularization activity provided valuable information for recurrence diagnostic of WHO grade 3 glioma.

**Supplementary Information:**

The online version contains supplementary material available at 10.1007/s00234-021-02740-9.

## Introduction

Tumor recurrence represents an inevitable event in the course of disease of most patients with WHO grade 3 glioma [1] and poses a major diagnostic and therapeutic challenge as recurrent WHO grade 3 glioma which are commonly detected at an advanced stage limiting existing treatment strategies. Gross-total resection of WHO grade 3 glioma recurrence has been associated with significantly prolonged overall survival compared with subtotal resection or non-surgical therapeutics [2]. With this in mind, early and reliable detection of WHO grade 3 glioma recurrence would allow more radical repeat resection of the smaller tumor promoting an increased survival.

Most studies assessed both glioblastoma (WHO grade 4) and WHO grade 3 glioma as malignant or high-grade brain tumors, and conclusions for the latter are based on extrapolations from these data. However, WHO grade 3 glioma, especially with a mutation of the isocitrate dehydrogenase (IDH) gene, differs prognostically from glioblastomas which are dominated by the IDH-wildtype [1]. Furthermore, only few number of in-human studies focused exclusively on patients with WHO grade 3 glioma; in particular, this holds true for recurrent WHO grade 3 glioma.

In general, treatment monitoring and recurrence detection of WHO grade 3 glioma are essentially based on MRI techniques. However, even advanced MRI approaches including measures of perfusion in combination with the updated criteria from the Response Assessment in Neuro-Oncology (RANO) group [3, 4] are limited to reliably detect WHO grade 3 glioma recurrence. This ambiguity requires additional follow-up examinations associated with loss of valuable time and further tumor progression or malignant transformation to glioblastoma.

Physiological MRI (phyMRI) approaches including vascular architecture mapping (VAM) and quantitative blood oxygenation level–dependent (qBOLD) imaging has been proposed to obtain deeper insight into the brain tumors pathophysiology such as neovascularization activity [5–7] and tissue oxygen tension (i.e., hypoxia) [8], respectively. The known physiological connection between neovascularization and tissue hypoxia mainly drives the rationale for the combination of these two MRI methodologies. VAM is based on the different sensitivity of gradient-echo (GE) and spin-echo (SE) MRI to magnetic susceptibility [5–7] leading to the fact that conventional GE perfusion MRI is dominated by larger arterioles and venules (vessel diameter > 20 μm) [5]. In contrast, SE perfusion MRI exhibits peak sensitivity to the microvasculature (capillaries and small vessels around 10 μm in diameter) [5]. The VAM approach [9] provides a framework for combined evaluation of GE and SE perfusion MRI data resulting in parametric maps for microvascular density, size, and type, respectively. It is worth noting that neovascularization at an early stage is clearly dominated by very thin vascular structures, which are consequently hardly detectable with GE perfusion MRI used in conventional MRI (cMRI) protocols. To date, assessment of tissue oxygen status is currently used only as a research tool.

Therefore, we hypothesized that phyMRI offers the capability to detect pathophysiological changes in the early developmental stage of IDH-mutated WHO grade 3 glioma recurrence. The purpose of this study was to evaluate the usefulness of phyMRI including VAM and qBOLD imaging for the monitoring of patients with WHO grade 3 glioma after standard of care therapy. We investigated the diagnostic performances for WHO grade 3 glioma recurrence detection and quantitatively analyzed the MRI-assessed pathophysiological features of early- and progressed-stage WHO grade 3 glioma recurrence.

## Materials and methods

The institutional review boards of the of the Lower Austrian Provincial Government and the Friedrich-Alexander University (FAU) Erlangen-Nürnberg approved both the prospective data acquisition and the retrospective data analysis of this study. Written informed consent in accordance with the ethical standards of the Declaration of Helsinki 1975 and its later amendments was obtained from all enrolled patients.

### Patient selection

A prospectively populated institutional database was searched for patients with WHO grade 3 gliomas (astrocytoma WHO grade 3 and oligodendroglioma WHO grade 3) who were treated with maximal safe and radical resection, radiotherapy, and concomitant and adjuvant chemotherapy with temozolomide [10] according to the European Organization for Research and Treatment of Cancer (EORTC) protocol and received MR examinations with our MRI study protocol between February 2016 and September 2020. Inclusion criteria were as follows: (**i**) aged ≥ 18 years; (**ii**) histopathologically confirmed WHO grade 3 glioma based on the WHO grading system as initial diagnosis; (**iii**) no previous diagnosis of WHO grade 3 glioma recurrence; (**iv**) no additional anti-glioma treatment but the standard of care (i.e., no antiangiogenic therapy, etc.); (**v**) MRI data acquisition was performed with the study protocol; and (**vi**) cMRI data were evaluated by at least two board-certified radiologists in consensus based on the updated RANO criteria [3, 4].

### MRI data acquisition

MRI data acquisition was performed on a clinical 3 Tesla scanner (Trio, Siemens, Erlangen, Germany) equipped with the standard 12-channel head coil. Follow-up MRI examinations were carried out every 3–6 months or on an unscheduled basis in case of clinical signs of tumor recurrence. The cMRI protocol for the diagnosis of brain tumors in clinical routine included (**i**) an axial fluid-attenuated inversion-recovery (FLAIR) sequence; (**ii**) an axial diffusion-weighted imaging sequence; (**iii**) pre- and post-contrast-enhanced (CE) high-resolution three-dimensional T1-weighted magnetization-prepared rapid acquisition with gradient-echo sequences; and (**iv**) a GE dynamic susceptibility contrast (DSC) perfusion MRI sequence during administration of 0.1 mmol/kg-bodyweight gadoterate meglumine (Dotarem, Guerbet, Roissy CdG, France) at a rate of 4 ml/s using a MR-compatible injector (Medrad, Volkach, Germany). A 20-ml bolus of saline was injected subsequently at the same rate. The parameters of the cMRI sequences are summarized in Table 1.

**Table 1:** Sequence parameters of the MRI study protocol

	Conventional MRI (cMRI) sequences				Physiological MRI sequences		
	FLAIR	MPRAGE	DWI	GE-DSC	SE-DSC	T_2_ mapping	T_2_* mapping
In-plane resolution	0.45 × 0.45	1.0 × 1.0	1.2 × 1.2	1.8 × 1.8	1.8 × 1.8	1.8 × 1.8	1.8 × 1.8
Slice thickness [mm]	3.0	1.0	4.0	4.0	4.0	4.0	4.0
Number of slices	48	176	29	29	29	29	29
TR [ms]	5000	2100	5300	1740	1740	3260	1210
TE [ms]	460	2.3	98	22	33	13–104 ms	5–40 ms
Flip angle* [°]	120	12	90	90	90	90	90
GRAPPA	2	2	2	2	2	2	2
Other	TI = 1800 ms		b = 0 and 1000 s/mm^2^	60 dynamic volumes	60 dynamic volumes	8 echoes	8 echoes

^*^Flip angle means the angle of excitation. Refocusing angles were 180° for all sequences with a SE scheme, i.e., FLAIR, DWI, SE-DSC, and T2 mapping

Abbreviations: *FLAIR*, fluid-attenuated inversion-recovery; *MPRAGE*, magnetization-prepared rapid acquisition with gradient-echo sequence for contrast-enhanced T1-weighted MRI; *DWI*, diffusion-weighted imaging; *GE-DSC*, gradient-echo dynamic susceptibility contrast perfusion MRI; *SE-DSC*, spin echo dynamic susceptibility contrast perfusion MRI; *GRAPPA*, parallel imaging using generalized autocalibrating partially parallel acquisition

The VAM approach [9] was used for MR-based assessment of microvascular architecture and neovascularization activity which required a DSC perfusion MRI sequence in combination with a SE echo-planar imaging read out. This SE-DSC sequence used the same parameters and contrast agent injection protocol as described for the routine GE-DSC perfusion MRI (Table 1). The first DSC MRI was obtained by the SE-DSC technique since SE-DSC perfusion MRI is less sensitive to contrast agent leakage [11]. Our strategies to minimize patient motion and differences in time to first-pass peak were described previously [9, 12].

The multiparametric qBOLD approach [8] was used for MR-based assessment of tissue oxygen metabolism which required a multi-echo GE sequence and a multi-echo SE sequence for mapping of the transverse relaxation rates R_2_* (= 1/T_2_*) and R_2_ (= 1/T_2_), respectively. All experimental sequences for VAM and qBOLD had the same identical slice position and geometric parameters (voxel size, number of slices, etc.) as used for the routine GE-DSC perfusion sequence. The additional data acquisition time for the VAM (SE-DSC perfusion, 2 min) and qBOLD sequences (R_2_* and R_2_-mapping, 1.5 and 3.5 min) was 7 min.

### MRI data processing and quantitative data analysis

Processing of cMRI, VAM, and qBOLD data as well as calculation of MRI biomarkers was performed with custom-made MATLAB (MathWorks, Natick, MA) software. Details of the MRI data processing pipeline were published previously [9, 12–14] and are described detailed in the supplementary material (including Supplementary Fig. 1). The procedure resulted in the MRI biomarker maps for apparent diffusion coefficient (ADC) representing microstructural density, cerebral blood volume (CBV), and microvascular cerebral blood volume (µCBV) representing macrovascular and microvascular perfusion, respectively, microvessel density (MVD), and vessel size index (VSI) as indicators for microvascular architecture and neovascularization activity represented by the microvessel type indicator (MTI), as well as oxygen metabolism including oxygen extraction fraction (OEF), cerebral metabolic rate of oxygen (CMRO_2_), and tissue oxygen tension (PO_2_). All nine biomarker maps are summarized at the bottom of Supplementary Fig. 1.

The regions of interest (ROIs) were manually defined by experienced radiologist, neurosurgeon, and MR physicist in consensus to cover areas of contrast enhancement on CE T1-weighted images suspected as tumor recurrence. Additional ROIs were positioned in contralateral normal brain, which were used as internal reference. The mean values for all nine MRI biomarkers were calculated for the ROIs. Patients were assigned to subgroups depending on their classification results: true positive (TP), false positive (FP), true negative (TN), and false negative (FN) results for detection of WHO grade 3 glioma recurrence. The phyMRI biomarker thresholds of a previous study [12] that investigated untreated WHO grade 3 glioma were used for recurrence prediction. The parameters for diagnostic performance (sensitivity, specificity, accuracy, and precision) were calculated from these data. Additionally, the total tumor volumes were determined on CE T1-weighted MRIs for patients with recurrent WHO grade 3 glioma.

### Statistical analysis

Statistical analyses were performed using R (version 3.6.3, R Foundation, Vienna, Austria) and SPSS (version 21, IBM, Chicago, IL, USA). Differences in imaging biomarkers between subgroups of patients were determined using the one-way analysis of variance (ANOVA) method. The Tukey test was used as post hoc procedure to be consistent with the assumption that homogeneity of variance was met and for correction for multiple comparisons. Homogeneity of variance was verified using the Levene’s test. When the assumption of homogeneity of variances was violated, Welch’s ANOVA in combination with the Games-Howell post hoc test was used. Intraindividual differences in imaging biomarker values between lesions and contralateral normal brain as well as in tumor volume between follow-up examinations were compared using a Wilcoxon signed-rank test. Significance of differences in tumor volume between patient subgroups was calculated using a Mann–Whitney *U* test. The area under the receiver operating characteristic (ROC) curve (AUC) was calculated for each MRI biomarker to determine the diagnostic performance for WHO grade 3 glioma recurrence detection. *P* values less than 0.05 were considered to indicate significance after Bonferroni correction for multiple comparisons.

## Results

### Patient characteristics

**Fig. 1:**
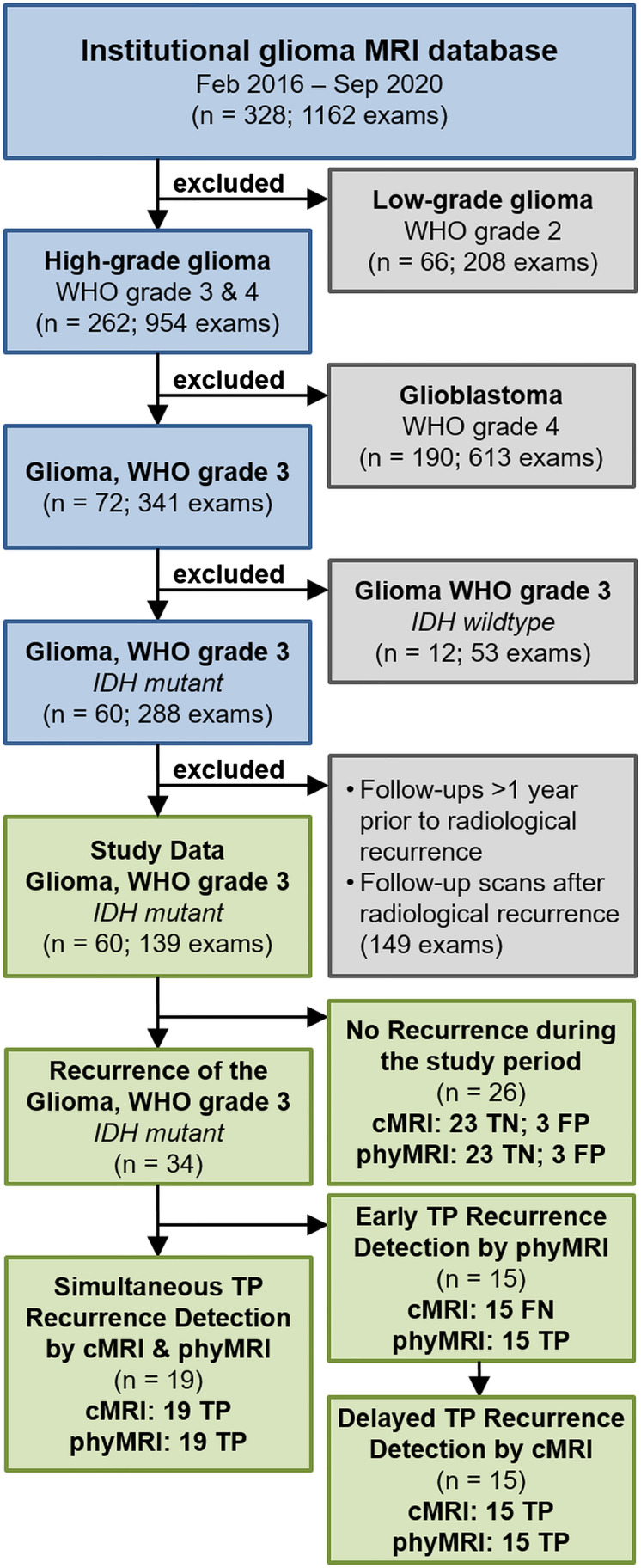
The upper part of the flow diagram summarizes the patient selection protocol including exclusion criteria as well as the number of patients (n) and MRI examinations (exams), respectively. The lower part visualizes the subgroups of patients analyzed in the study (green boxes)

The institutional database contained almost 1200 MRI examinations using the study MRI protocol in 328 patients with glioma. A flow diagram for patient selection is depicted in the upper part of Fig. 1. Patients with low-grade glioma (WHO grade 2, n = 66) or glioblastoma (WHO grade 4, n = 190) were excluded. Of the remaining 72 patients with WHO grade 3 glioma, 12 were excluded for IDH-wildtype status. Among the 60 included patients (34 men; mean age 48.4 ± 11.9 years), there were 35 patients suffering from an astrocytoma WHO grade 3 and 25 with an oligodendroglioma WHO grade 3. From these 60 patients, 34 patients showed recurrence of the WHO grade 3 glioma; 20 patients suffered from a recurrent astrocytoma and 14 from a recurrent oligodendroglioma WHO grade 3. In 20 patients, recurrence was established by histopathology of tissue samples obtained during repeat craniotomy and in 14 patients by MRI follow-up, respectively. The residual 26 patients showed no signs for WHO grade 3 glioma recurrence during the study period (lower part of Fig. 1); from these, 15 patients initially had an IDH-mutant astrocytoma and 11 patients an oligodendroglioma WHO grade 3. Patient characteristics and clinical data are summarized in Table 2.

**Table 2:** Patient characteristics and clinical data of the 60 patients

Characteristic	Value
Sex	
Female	26 (43.3)
Male	34 (56.7)
Mean age (years) ± standard deviation	48.4 ± 11.9
Age range (years)	21.1–76.4
Age interquartile range (years)	40.0–57.9
WHO classification	
Astrocytoma, WHO grade 3, *IDH mutant*	35 (58.3)
Oligodendroglioma, WHO grade 3, *IDH mutant, 1p19q codeleted*	25 (41.7)
Recurrence of the glioma WHO grade 3	34 (56.7)
Astrocytoma, WHO grade 3, *IDH mutant*	20 (58.8)
Oligodendroglioma, WHO grade 3, *IDH mutant, 1p19q codeleted*	14 (41.2)
Mean time to radiological recurrence after resection (months)	34.8 ± 27.5
Astrocytoma, WHO grade 3, *IDH mutant*	29.8 ± 24.3
Oligodendroglioma, WHO grade 3, *IDH mutant, 1p19q codeleted*	41.8 ± 31.1
Range of time to radiological recurrence after resection (months)	9.1–98.4
Astrocytoma, WHO grade 3, *IDH mutant*	9.1–94.3
Oligodendroglioma, WHO grade 3, *IDH mutant, 1p19q codeleted*	10.6–98.4
Treatment of the recurrent WHO grade 3 glioma	
Repeat craniotomy	20 (58.8)
Repeat radiation therapy or repeat combined radio-chemotherapy	6 (17.7)
Temozolomide rechallenge	4 (11.8)
Second line monotherapy with Bevacizumab	3 (8.8)
Second line monotherapy with Nivolumab	1 (2.9)
Malignant transformation to glioblastoma WHO grade 4 at recurrence	7 (11.7)
Initial astrocytoma, WHO grade 3, *IDH mutant*	7 (100)

Unless otherwise specified, data are numbers of patients, with percentages in parentheses. The values for the World Health Organization (WHO) classification listed here are based on results of fluorescence in situ hybridization testing for 1p/19q assignment. *IDH*, isocitrate dehydrogenase

### Detection of WHO grade 3 glioma recurrence with cMRI and phyMRI

**Fig. 2:**
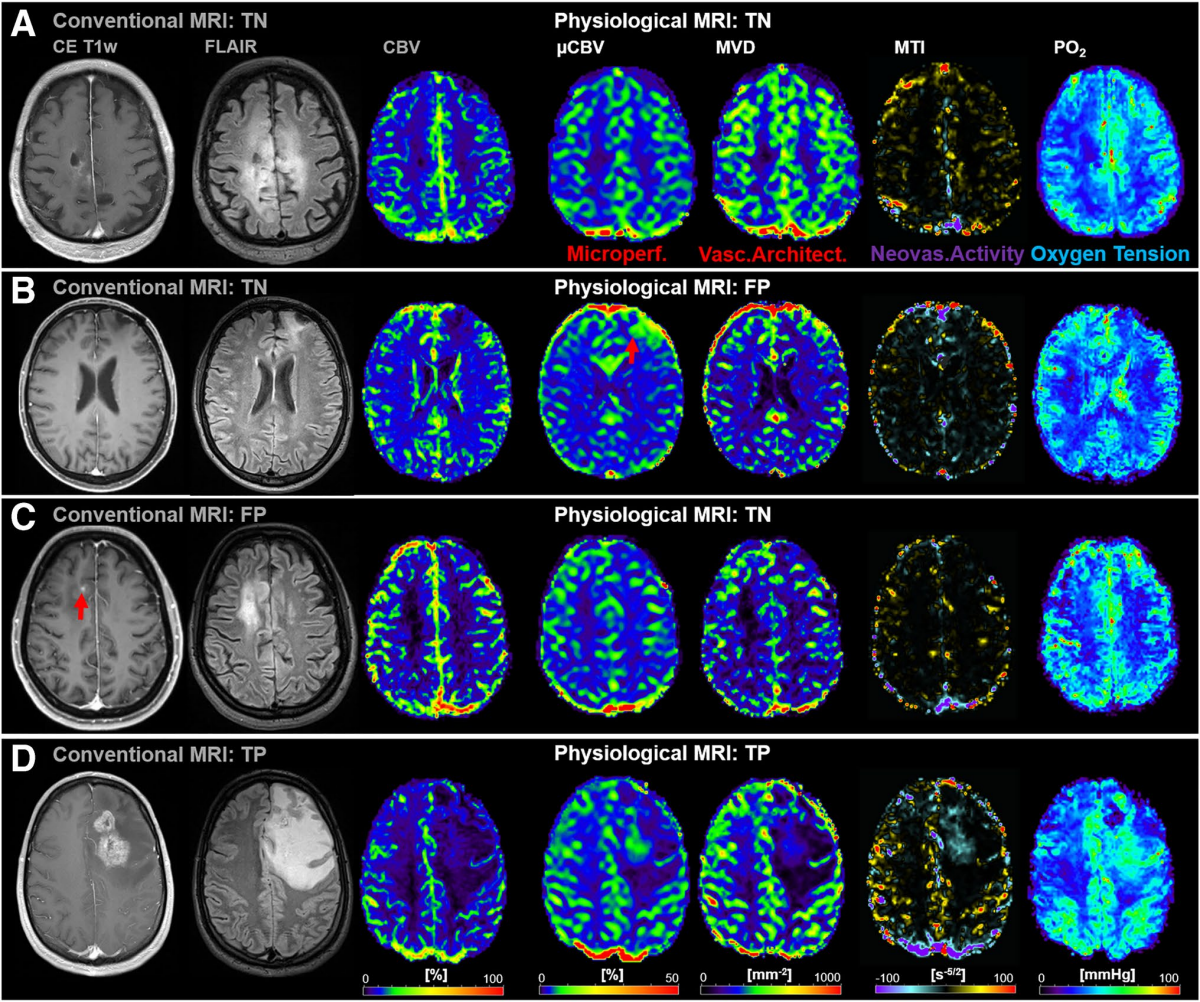
**A** TN finding by both cMRI and phyMRI. The cMRI and phyMRI data of a 61-year-old male patient revealed no evidence for recurrence of the astrocytoma WHO grade 3. This patient showed no evidence for recurrence in the next follow-up examination 6 months later. **B** FP finding by phyMRI. The phyMRI biomarker map for µCBV of a 52-year-old male patient showed increased microvascular perfusion in the vicinity of the resection cavity which was (incorrectly) interpreted as recurrence of the oligodendroglioma WHO grade 3. Noteworthy, MTI showed no evidence for neovascularization activity and PO_2_ restored or increased oxygen levels. The cMRI showed no evidence for recurrence, i.e., were TN, because the next two follow-up examination 6 and 12 months later also showed no evidence for recurrence. The area with increased µCBV was not visible in the next follow-ups. **C** FP finding by cMRI. The CE T1w MRI of a 46-year-old female patient showed a new contrast enhancement in the vicinity of the resection cavity (standard therapy was 4.2 years ago) which was (incorrectly) interpreted as recurrence of the oligodendroglioma WHO grade 3. The phyMRI data showed no evidence for recurrence, i.e., were TN, because the next four follow-up examination also showed no evidence for recurrence. The CE area was not visible in the next follow-ups. **D** Simultaneous TP detection of WHO grade 3 glioma recurrence in both cMRI and phyMRI. cMRI including anatomical sequences (CE T1w and FLAIR) and macrovascular perfusion (CBV), as well as phyMRI biomarker maps of microvascular perfusion (µCBV), microvessel density (MVD), neovascularization activity (MTI), and tissue oxygen tension (PO_2_) of a 31-year-old male patient clearly demonstrated recurrence of an astrocytoma WHO grade 3. This patient received a temozolomide rechallenge

In the 26 patients who showed no recurrence during the study period, cMRI and phyMRI consistently revealed no recurrence (i.e., a TN result) in 20 patients. An illustrative case for TN findings by both cMRI and phyMRI is depicted in Fig. 2A. However, FP results were found in three patients for cMRI and phyMRI, respectively. In all six FP cases, no recurrence treatment was correctly initiated because clinical parameters showed no evidence for recurrence. The subsequent follow-up examinations of these patients (two to six examinations over 12–45 months) actually revealed no evidence for recurrence.

Figure 2B illustrates a patient with a FP finding by phyMRI who showed signs of capillary hyperperfusion (increased µCBV, red arrow) but no obvious macrovascular hyperperfusion (CBV), contrast enhancement, or neovascularization activity. The subsequent two follow-up examinations 6 and 12 months later, respectively, showed no signs for recurrence. Therefore, the initial findings were TN for cMRI. In Fig. 2C, a patient with a FP finding by cMRI (new small contrast enhancement, red arrow) is depicted. However, neither macro- and microvascular perfusion (CBV and µCBV) nor microvascular architecture (MVD) and neovascularization activity (MTI) showed signs for recurrence during the subsequent six follow-ups, six over a time period of 45 months. Therefore, the initial findings were interpreted as TN for phyMRI.

**Fig. 3:**
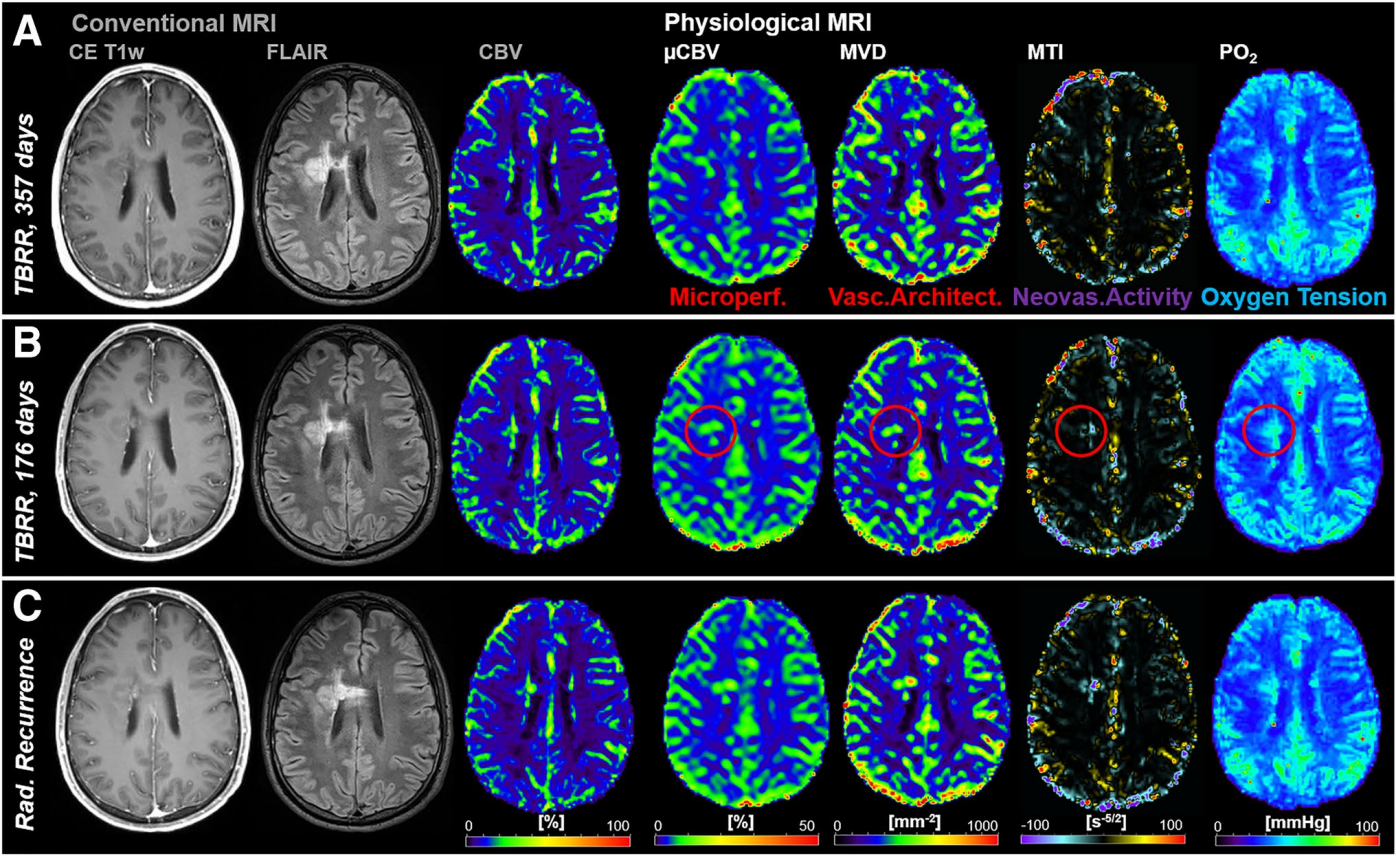
Early TP WHO grade 3 glioma recurrence detection by phyMRI data. Three consecutive follow-up examinations using cMRI including anatomical sequences (CE T1w and FLAIR) and macrovascular perfusion (CBV) as well as phyMRI biomarker maps of microvascular perfusion (µCBV), microvessel density (MVD), microvessel type indicator (MTI), and tissue oxygen tension (PO_2_) of a 34-year-old male patient who received standard therapy of an astrocytoma WHO grade 3 one year ago. **A** At 357 days before radiological recurrence was detected (TBRR = time before radiological recurrence), both cMRI and phyMRI data showed no evidence for recurrence. **B** 181 days later (TBRR = 176 days), cMRI was interpreted to show no evidence for recurrence, but phyMRI biomarker data for microvascular perfusion (µCBV) and architecture (MVD) as well as neovascularization activity (MTI) and tissue oxygen tension (PO_2_) revealed signs for recurrence of the astrocytoma WHO grade 3 (red circles). **C** At the subsequent follow-up examination, the patient showed clear signs of recurrence in cMRI data and progression of the recurrent astrocytoma WHO grade 3 in phyMRI data, respectively. The tumor volume has increased 7.7-fold from 0.3 to 2.3 cm^3^ within these 176 days. The patient received radiation therapy

**Fig. 4:**
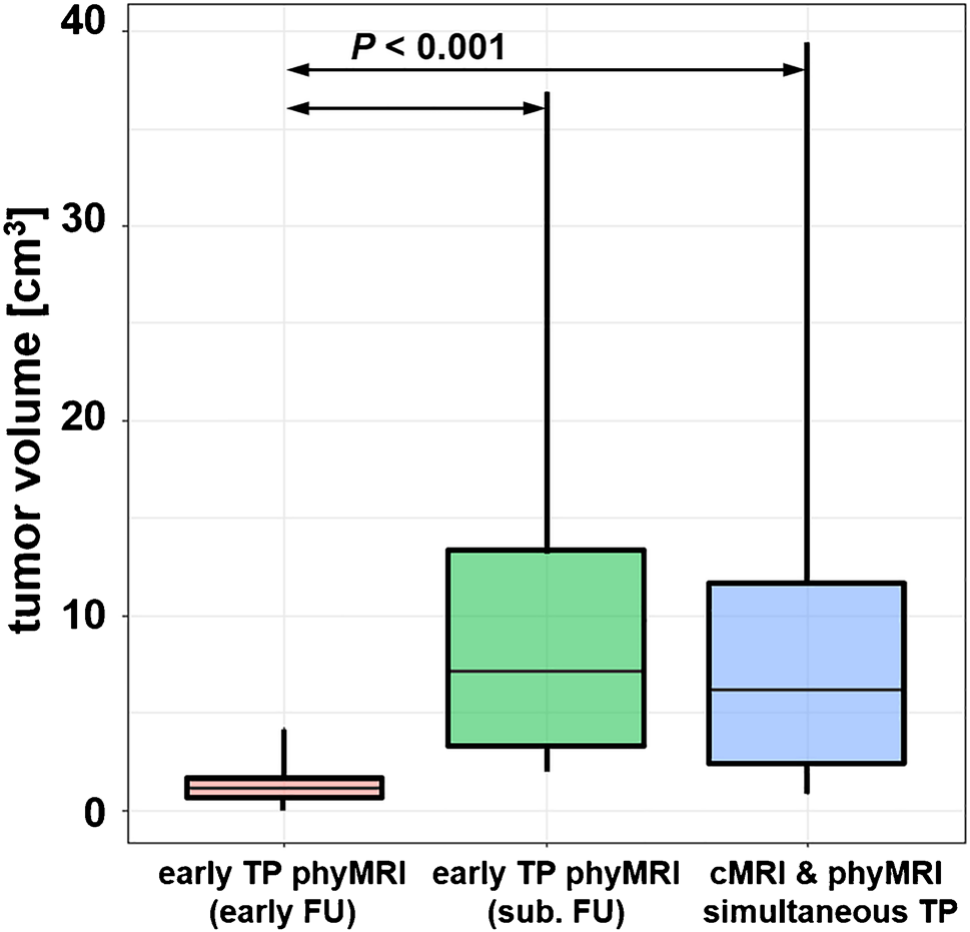
Tumor volumes for the patient subgroups with early TP findings by phyMRI biomarker data and a FN finding by cMRI data (“early TP phyMRI (early FU)”; red box-whisker plot) at the initial follow-up examination. The corresponding tumor volumes of the same patients at the subsequent follow-up with delayed TP results in cMRI (“early TP phyMRI (sub. FU)”; green box-whisker plot) is depicted in the middle. The blue box-whisker plot on the right summarizes the tumor volumes for the patient subgroup with simultaneous TP findings by both cMRI and phyMRI data. Abbreviations: FU, follow-up; sub. FU, subsequent follow-up

In the 34 patients with recurrent WHO grade 3 glioma during the study period, recurrence was detected simultaneously TP by both cMRI and phyMRI data of the same follow-up examination in 19 of the 34 patients (55.9%; 12 astrocytoma, 7 oligodendroglioma). This patient subgroup was termed “simultaneously TP,” and an illustrative case is depicted in Fig. 2D. In the remaining 15 patients with recurrent WHO grade 3 glioma (44.1%; 8 astrocytoma, 7 oligodendroglioma), recurrence was detected earlier by phyMRI data of follow-up examinations whose cMRI data showed no evidence for recurrence, i.e., cMRI was FN (lower part of Fig. 1). This subgroup was termed “early TP phyMRI.” Recurrence, however, was detected by the cMRI data of the subsequent follow-up examination. An illustrative case is presented in Fig. 3. In this patient, recurrence of the WHO grade 3 glioma was detected by phyMRI (Fig. 3B) 176 days prior to recurrence detection by cMRI (Fig. 3C). The averaged time differences between the early and the subsequent follow-up examination for all 15 patients of the “early TP phyMRI” subgroup were 157 ± 59 days (range, 68–271 days). During this time period, the tumor volumes increased statistically significant (*P* < 0.001) from 1.5 ± 1.2 cm^3^ (0.3–4.3 cm^3^) to 11.1 ± 11.0 cm^3^ (2.1–36.9 cm^3^; Fig. 4). The degree of tumor volume increase ranged between doubling to 17-fold increase (7.4-fold on average). For astrocytoma WHO grade 3, the tumor volume increase ranged between 2.9- and 16.8-fold (7.6-fold on average) and for oligodendroglioma WHO grade 3 between 2.0- and 11.1-fold (7.1-fold on average). There were no FN results for phyMRI.

### Quantitative evaluation of imaging biomarkers

The values of the cMRI biomarkers (ADC and macrovascular CBV) and the phyMRI biomarkers (µCBV, MVD, VSI, MTI, OEF, CMRO_2_, and PO_2_) for the subgroups of patient with and without recurrence of a WHO grade 3 glioma are summarized in Table 3. We investigated the differences in biomarker values between the patient subgroups in order to find reasons for the FN and FP findings of both cMRI and phyMRI.

**Table 3:** Imaging biomarker values of cMRI and phyMRI for the subgroups of patients

	No recurrence			Recurrence			cNB
	Both TN	FP phyMRI	FP cMRI	Simultan. TP	early TP phyMRI		
					early FU (FN cMRI)	subsequ. FU	
ADC [mm^2^/s]	1.24 ± 0.31 0.73–1.77	1.07 ± 0.40 0.56–1.50	2.14 ± 0.65 1.62–2.87	1.37 ± 0.34 0.69–1.89	1.44 ± 0.33 0.90–1.92	1.25 ± 0.25 0.92–1.79	0.83 ± 0.10 0.69–1.10
CBV [%]	4.7 ± 2.0 2.0–8.9	10.6 ± 5.8 6.3–19.0	5.0 ± 4.4 2.0–10.0	19.9 ± 9.7 7.3–40.0	6.5 ± 1.9 3.7–10.6	16.8 ± 5.5 7.4–24.7	6.2 ± 1.6 3.0–8.8
µCBV [%]	2.4 ± 0.7 1.2–3.3	5.5 ± 1.8 3.2–7.3	1.8 ± 0.6 1.4–2.5	7.6 ± 2.2 3.9–13.7	3.6 ± 1.3 1.5–5.7	7.4 ± 2.0 3.2–10.2	3.2 ± 0.8 1.6–4.7
MVD [mm^−2^]	234 ± 41 147–308	283 ± 111 122–368	107 ± 24 82–130	422 ± 160 221–759	216 ± 93 80–349	379 ± 141 190–577	219 ± 57 108–317
VSI [µm]	53 ± 11 37–75	67 ± 36 23–109	66 ± 67 21–143	78 ± 27 41–132	61 ± 15 31–75	65 ± 21 33–100	45 ± 15 18–76
MTI [s^−5/2^]	− 0.7 ± 1.1 − 2.9–1.1	− 6.9 ± 4.2 − 10.7–1.9	− 0.4 ± 0.7 − 1.2–0.1	− 29 ± 21 − 72– − 9	− 4.6 ± 5.9 − 17.7– − 0.4	− 31 ± 11 − 48– − 5.4	0.0 ± 1.3 − 2.3–2.7
OEF [%]	32 ± 6 24–45	44 ± 16 28–64	54 ± 26 29–81	21 ± 16 5–69	38 ± 14 24–69	25 ± 12 13–52	36 ± 9 22–59
CMRO_2_ [µM/100 g∙min]	80 ± 24 42–120	162 ± 62 74–211	89 ± 48 56–144	120 ± 84 32–342	136 ± 55 46–197	123 ± 52 53–194	101 ± 26 60–153
PO_2_ [mmHg]	41 ± 9 24–57	26 ± 10 18–41	34 ± 15 20–50	55 ± 23 21–88	30 ± 15 5–56	52 ± 17 24–79	35 ± 8 22–54
n	20	3	3	19	15		60

Both IDH-mutant astrocytoma WHO grade 3 and oligodendroglioma WHO grade 3 were included in the calculation of the biomarker values. Abbreviations: *CBV*, cerebral blood volume in macrovasculature; *µCBV*, CBV in microvasculature; *MVD*, microvessel density; *VSI*, vessel size index; *MTI*, microvessel type indicator; *n*, patient number in the subgroup; *both TN*, true negative recurrence detection in cMRI and VAM; *FP phyMRI*, false positive recurrence detection in physiological MRI; *FP cMRI*, false positive recurrence detection in cMRI; *Simultan. TP*, simultaneous and correct glioma WHO grade 3 recurrence detection by both cMRI and phyMRI data; *early TP phyMRI*, early true positive recurrence detection in phyMRI; *early FU*, the early follow-up in the “early TP phyMRI” subgroup with a FN finding in the cMRI data; *subsequ. FU*, the subsequent follow-up in the “early TP phyMRI” subgroup with a delayed TP finding in the cMRI data; cNB, contralateral normal brain

In patients with a FN finding by cMRI, i.e., an early TP phyMRI finding (fifth column in Table 3), macrovascular CBV was significantly lower (*P* < 0.001) compared to both subgroups with TP findings in cMRI (fourth and sixth columns in Table 3) but was not significantly different to CBV values in TN findings (*P* = 0.113) and in contralateral normal brain (cNB; *P* = 0.649). This was a quantitative confirmation for the absence of macrovascular hyperperfusion in the early recurrence of IDH-mutant WHO grade 3 glioma. The ADC values for this patient subgroup with FN cMRI findings, however, were not significantly different from both subgroups with TP findings in cMRI (vs. fourth column “simultan. TP,” *P* = 0.985; vs. sixth column “subsequent FU,” *P* = 0.590). Moreover, there was also no significant difference to the ADC values for the TN findings. This is related with the known low specificity of ADC changes in WHO grade 3 glioma recurrence as shown previously [15].

In patients with FP finding in cMRI (third column in Table 3), CBV values were significantly lower compared to both subgroups with TP findings in cMRI (vs. fourth column “simultan. TP,” *P* = 0.001; vs. sixth column “subsequent FU,” *P* = 0.029) and were not significantly different to CBV in cNB (*P* = 0.916). This indicates that the quantitative analysis of CBV was insufficiently considered during diagnosis of these three cases.

In patients with FP finding in phyMRI (second column in Table 3), microvascular perfusion (µCBV) was not significantly different compared to TP findings (vs. fourth column “simultan. TP,” *P* = 0.390; vs. fifth column “early TP phyMRI”; “early FU,” *P* = 0.574; vs. sixth column “early TP phyMRI”; “subsequent FU,” *P* = 0.640) but was significantly increased compared to TN findings (first column, *P* = 0.015). Interestingly, neovascularization activity (MTI) showed reverse findings: significant differences between FP and late TP findings in phyMRI (vs. fourth column “simultan. TP,” *P* = 0.022; vs. sixth column “early TP phyMRI”; “subsequent FU.” *P* = 0.013) but not compared to TN findings (*P* = 0.241). This was a quantitative confirmation that there was indeed microvascular hyperperfusion but without significant neovascularization activity, possibly related to gliosis. Like neovascularization activity (MTI), tissue oxygen tension (PO_2_) showed significant differences between FP findings in phyMRI and late TP findings (vs. fourth column “simultan. TP,” *P* = 0.024; vs. sixth column “early TP phyMRI”; “subsequent FU,” *P* = 0.041) but not compared to TN findings (*P* = 0.265). This was an indication for the known physiological relation between hypoxia and neovascularization activity. Finally, the phyMRI biomarkers MVD, VSI, OEF, and CMRO_2_ as well as the cMRI biomarker ADC were not significantly different between the FP and TN findings as well as compared to the TP findings. This was an indication for a low specificity of these parameters for WHO grade 3 glioma recurrence detection.

In summary, quantitative analysis revealed that microvascular perfusion (µCBV) and neovascularization activity (MTI) were best suited for the detection of WHO grade 3 glioma recurrence. The biomarker values for astrocytoma WHO grade 3 and oligodendroglioma WHO grade 3 are separately summarized in the Supplementary Tables 1 and 2, respectively.

### Diagnostic performance of MRI biomarkers for WHO grade 3 glioma recurrence detection

**Fig. 5:**
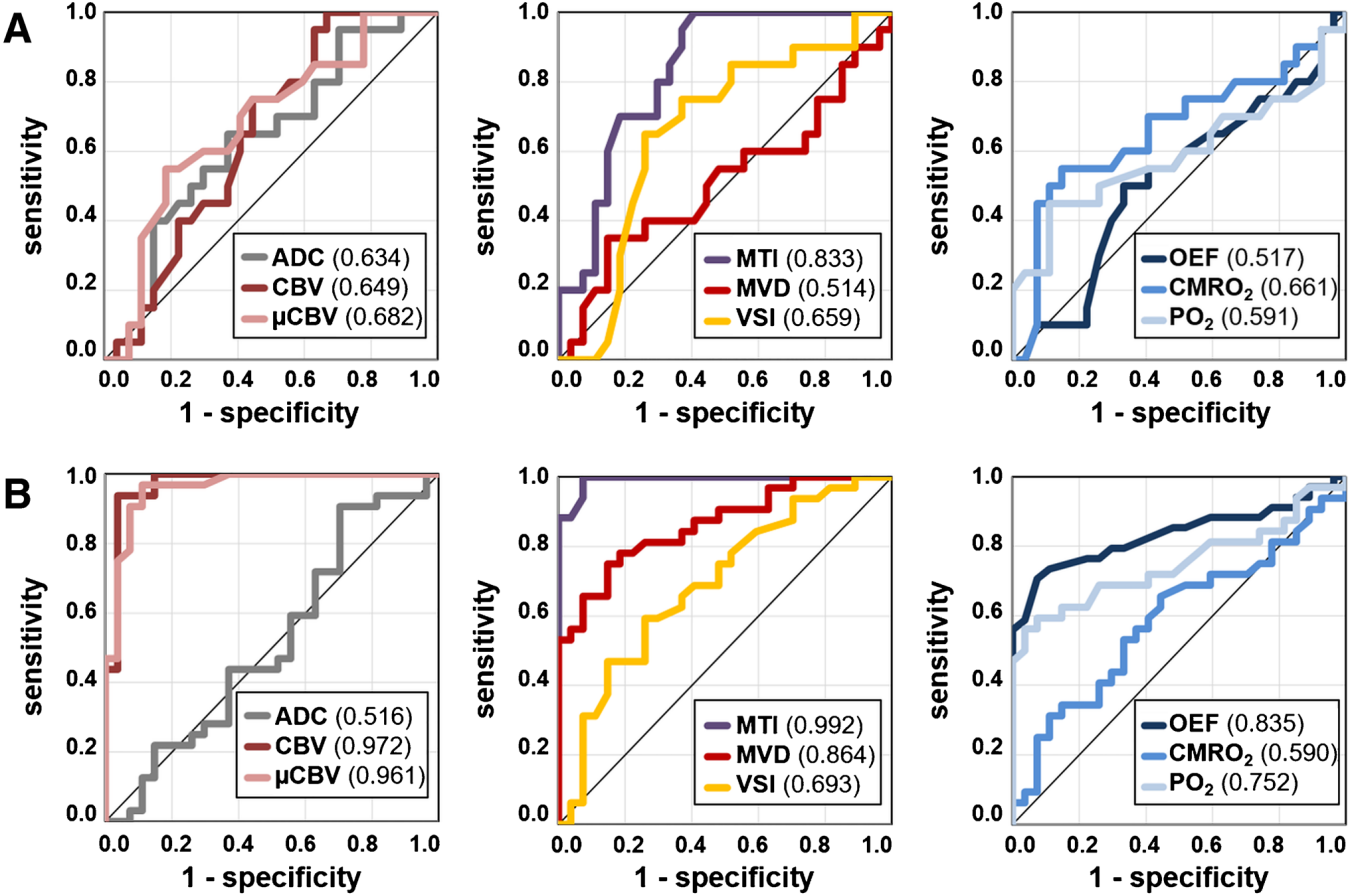
Receiver operating characteristic (ROC) curve analyses illustrate the diagnostic performance for WHO grade 3 glioma recurrence detection of cMRI (ADC and CBV) and phyMRI biomarkers (µCBV, MTI, MVD, VSI, OEF, CMRO_2_, and PO_2_). **A** At early detection by phyMRI (early follow-up) as well as **B** at delayed detection by cMRI (subsequent follow-up) and simultaneously TP. For both, the phyMRI biomarker for neovascularization activity (MTI) had the highest area under the ROC curve (AUC, 0.833 and 0.992) for WHO grade 3 glioma recurrence detection. Note. – The individual AUC values are in the parenthesis in the image legends

Sensitivity, specificity, accuracy, and precision for early detection of WHO grade 3 glioma recurrence were 0.559, 0.885, 0.700, and 0.864 for cMRI and 1.0, 0.885, 0.950, and 0.919 for phyMRI, respectively. ROC curve analysis for early WHO grade 3 glioma recurrence detection (Fig. 5A) revealed highest diagnostic performance for MTI (AUC = 0.833) followed by five biomarkers with rather similar diagnostic performance: µCBV (0.682), CMRO_2_ (0.661), VSI (0.659), CBV (0.649), and ADC (0.634). PO_2_ (0.591), OEF (0.517), and MVD (0.514) showed inferior diagnostic performance for early WHO grade 3 glioma recurrence detection.

**Fig. 6:**
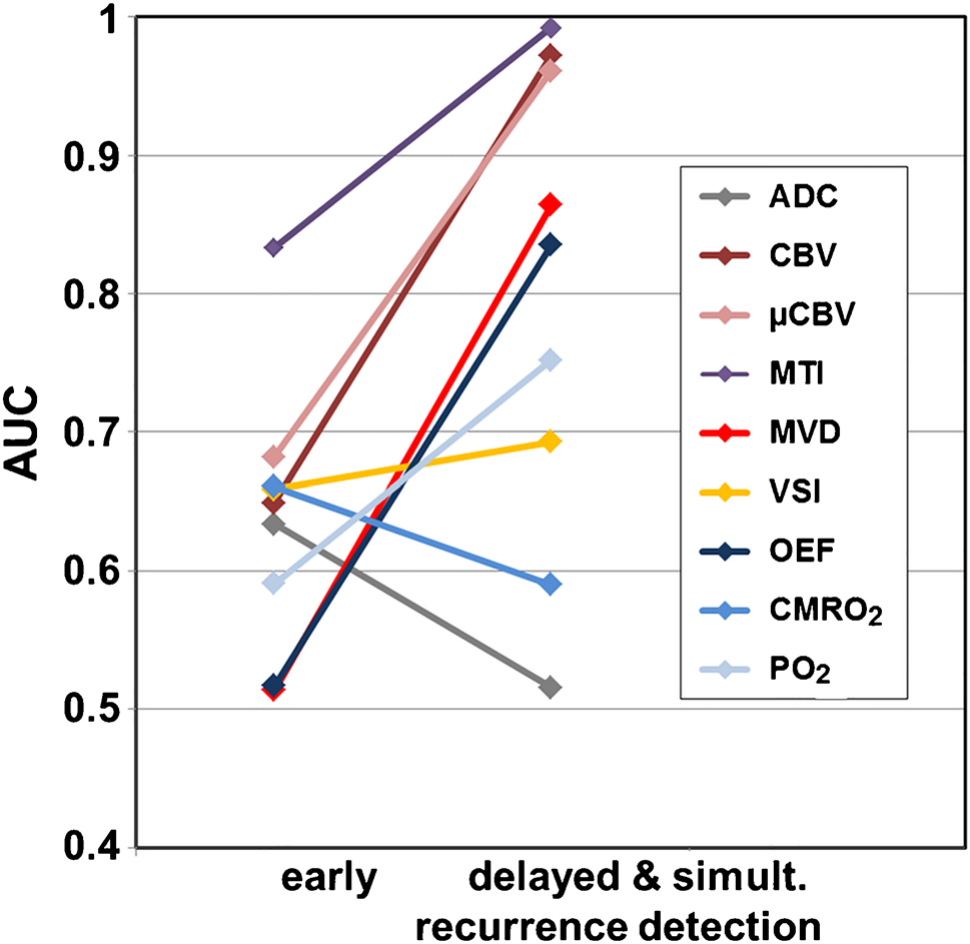
Changes in the area under the receiver operating characteristic (ROC) curve (AUC) between early recurrence detection and delayed and simultaneous detection of WHO grade 3 glioma recurrence. The associated ROC curves are depicted in Fig. 5

Diagnostic parameters for simultaneously TP and delayed TP cMRI were identical for both cMRI and phyMRI: sensitivity, 1.0; specificity, 0.885; accuracy, 0.950; and precision, 0.919. ROC curve analysis (Fig. 5B) revealed highest diagnostic performance for MTI (AUC, 0.992), CBV (0.972), and µCBV (0.961) followed by three biomarkers with intermediate diagnostic performance: MVD (0.864), OEF (0.835), and PO_2_ (0.752). VSI (0.693), CMRO_2_ (0.590), and ADC (0.516) showed inferior diagnostic performance for early WHO grade 3 glioma recurrence detection. Most of the MRI biomarker showed a strong to moderate increase in diagnostic performance from early TP phyMRI (early follow-up) to delayed TP cMRI (subsequent follow-up of the same patients) as well as simultaneously TP of both cMRI and phyMRI, except for ADC and CMRO_2_ which showed even a decrease in the AUC (Fig. 6).

A separate analysis of the diagnostic performance for astrocytoma and oligodendroglioma WHO grade 3 also revealed higher parameters for the biomarkers associated with neovascularization, perfusion, and tumor vasculature compared to oxygen metabolism or microstructural density. Details of the analysis are summarized in the Supplementary Results and Supplementary Fig. 2.

## Discussion

This study applied an extended MRI protocol including a phyMRI approach, which is fully compatible with the requirements of radiological routine diagnostics of glioma [9, 12, 13]. We searched for early alterations in macro- and microvascular perfusion, microvascular architecture, neovascularization activity, oxygen metabolism (including tissue hypoxia), and microstructural density that occurred during development of recurrence in patients with IDH-mutant WHO grade 3 glioma. Our main findings were threefold: (**i**) phyMRI enabled detection of early pathophysiological changes during development of WHO grade 3 glioma recurrence; (**ii**) early WHO grade 3 glioma recurrence showed microvascular (µCBV) but no macrovascular (CBV) hyperperfusion; and (**iii**) neovascularization activity (MTI) showed superior diagnostic performance compared with cMRI for both early as well as delayed detection of WHO grade 3 glioma recurrence.

Early and reliable detection of glioma recurrence is of high clinical importance and essential for personalized patient management targeting diagnosis as well as therapy. Several previous studies used diffusion and perfusion MRI, MR spectroscopy, and/or positron emission tomography and demonstrated the usefulness of various imaging biomarkers for prediction of recurrence and patient outcome [16–19]. Three comprehensive meta-analyses [20–22] investigated the diagnostic performance of these imaging techniques and biomarkers for recurrence detection of high-grade glioma and found values for sensitivity and specificity that are in accordance with our findings for late detection. However, these studies neither included data of earlier follow-up scans nor a separate analysis for IDH-mutant WHO grade 3 glioma.

Our phyMRI approach indicated pathophysiological alterations associated with WHO grade 3 glioma recurrence half a year earlier than radiological recurrence detection by cMRI. This was in line with previous published data concerning the phyMRI biomarkers in order to predict glioblastoma recurrence [23, 24]. In these studies as well as in the current study, the phyMRI biomarkers for neovascularization activity (MTI) and microvascular perfusion (µCBV) demonstrated the highest diagnostic performance for early recurrence detection. These observations support the fact that an initial step in high-grade glioma recurrence is an activation of neovascularization [25] driving to vascular proliferation and increased microvessel density to insure oxygen and nutrient supply of the tumor. Our current data revealed similar findings compared with previous publications which demonstrated that the microvascular structure [26, 27] and hypoxia [28] of gliomas are an important prognostic factor. The increase in tissue hypoxia (decrease in PO_2_) observed in the current study may in part reflect the known physiological relation between hypoxia and neovascularization activity [25] and may be an early sign of a switch from a non-hypervascularized highly infiltrative to a hypervascularized proliferative phenotype. Interestingly, OEF showed the second highest AUC values for both early and delayed detection of oligodendroglioma WHO grade 3 recurrence, unlike astrocytoma WHO grade 3. Otherwise, the differences between the tumor subgroups were less significant.

There are several limitations in our study. Two separate contrast agent injections were required for our VAM approach. However, the GE-DSC perfusion sequence is essential for clinical routine diagnosis, and this strategy ensures that it is kept unchanged regarding spatial and temporal resolution. Furthermore, this enables the acquisition of VAM data that cover the whole brain with high spatial resolution and sufficient signal-to-noise. Alternatively, the combined approach using a GE-SE-DSC perfusion sequence [29–31] does not meet the above-mentioned requirements for clinical routine diagnosis. Furthermore, the GE-SE-approach needs the use of at least a double dose [29–31]. Our multiparametric qBOLD approach is limited because it provides only an estimation of the oxygen metabolism with model-inherent restrictions. The model assumptions are that the system under investigation is in the static dephasing regime [32], whereby OEF is predominantly weighted to the medium sized and larger venules and provides an average blood oxygenation within the entire vasculature. Additionally, hemosiderin or protein accumulations, background gradients, white matter fiber orientation, and contrast agent leakage could bias the OEF estimation [33–35]. Finally, the number of patients with simultaneous TP (n = 19) and early TP in phyMRI findings (n = 15) was relatively small which was related to our rather strict inclusion/exclusion criteria (e.g., IDH mutation status, standard treatment only). Prospective studies evaluating the clinical usefulness of our phyMRI approach for WHO grade 3 glioma recurrence detection deserve further attention.

Conclusively, this study demonstrated that the targeted characterization of microvascular features and tissue oxygen tension provided clinically valuable insights relative to the early neovascularization activity during WHO grade 3 glioma recurrence which is complementary to and compatible with cMRI. With this in mind, our approach may be useful for more reliable, precise monitoring of patients suffering from WHO grade 3 glioma in order to detect early recurrence. Hence, the early forecasting of WHO grade 3 glioma recurrence may help to develop and implement personalized timely MRI-based decision-making addressed to targeted surgery and adjunctive therapeutics.

## Supplementary Information

Below is the link to the electronic supplementary material.

Supplementary file1 (DOCX 3750 kb)

## Abbreviations

cMRI: Conventional MRI
phyMRI: Physiological MRI
IDH: Isocitrate dehydrogenase
VAM: Vascular architecture mapping
qBOLD: Quantitative blood-oxygen-level-dependent
ADC: Apparent diffusion coefficient
CBV: Cerebral blood volume
µCBV: Microvascular cerebral blood volume
MVD: Microvessel density
VSI: Vessel size index
MTI: Microvessel type indicator
OEF: Oxygen extraction fraction
CMRO_2_: Cerebral metabolic rate of oxygen
PO_2_: Tissue oxygen tension
TP: True positive
FP: False positive
TN: True negative
FN: False negative
