# Supplementary material for: Physiological MRI of microvascular architecture, neovascularization activity, and oxygen metabolism facilitate early recurrence detection in patients with IDH-mutant WHO grade 3 glioma
Source: Neuroradiology. 2021 Jun 11;64(2):265–77. doi: 10.1007/s00234-021-02740-9 (PMC8789727; doi:10.1007/s00234-021-02740-9)
Supplement: Supplementary file 1 — Supplementary file1 (DOCX 3750 kb) [file 234_2021_2740_MOESM1_ESM.docx]

**SUPPLEMENTARY MATERIALS**

**MRI Data processing**

The VAM data processing (red lines in **Supplementary Figure 1**) consisted of five steps: (**i**) Correction for remaining contrast agent extravasation was performed as described previously [1–3]; (**ii**) fitting of the first bolus curves for each voxel of the GE- and SE-DSC perfusion MRI data with a previously described gamma-variate function [4], (**iii**) Calculation of the ∆R_2,GE_ versus (∆R_2,SE_)^3/2^ diagram [5], the so-called vascular hysteresis loop (VHL) [3, 6]. These data were subsequently used for (**iv**) calculation of maps for microvascular architecture including microvessel density (MVD), the vessel size index (VSI, i.e. microvessel radius) [7] as well as for neovascularization activity represented by the microvessel type indicator (MTI) [3]. For MVD and VSI, we used the following equations:

$\text{MVD =}\text{ }\frac{\text{Q}_{\text{max}}}{\text{b}}\text{∙}\left( \frac{\text{CBV}^{\text{2}}}{\text{4}\text{π}^{\text{2}}\text{∙ADC∙}{\bar{\text{R}}}^{\text{4}}} \right)^{\text{1}/\text{3}}$ and $\text{VSI = }\left( \frac{\text{CBV∙ADC∙}\text{b}^{\text{3}}}{\text{2π∙}\text{Q}_{\text{max}}^{\text{3}}} \right)^{\text{1}/\text{2}}$

with Q_max_ = max[∆R_2,GE_]/max[(∆R_2,GE_)^3/2^]; ADC = apparent diffusion coefficient which was calculated from the DWI data; $\bar{\text{R}}$ ≈ 3.0 μm is the mean vessel lumen radius and b is a numerical constant (b = 1.6781) [7]. MTI was defined as the area of the VHL signed with the rotational direction of the VHL, i.e. a clockwise VHL-direction was identified with a plus-sign, and a counter-clockwise VHL-direction was identified with a minus-sign [3]. In a final step (**v**) the map for the microvascular cerebral blood volume (μCBV) was calculated from the SE-DSC perfusion MRI data via a separate automatic identification of AIFs [8]. In summary, this resulted in the MRI biomarker maps of microstructural density (ADC), perfusion (CBV and µCBV), microvascular architecture (MVD and VSI), and neovascularization activity (MTI), respectively.

The qBOLD data processing (blue lines in **Supplementary Figure 1**) consisted of four steps: (**i**) Corrections for background fields of the R_2_*-mapping data [9] and for stimulated echos of the R_2_-mapping data [10]; (**ii**) Calculation of R_2_*- and R_2_-maps from the multi-echo relaxometry data; and (**iii**) of absolute cerebral blood volume (CBV) and flow (CBF) maps from the GE-DSC perfusion MRI data via automatic identification of arterial input functions (AIFs) [8, 11]. In the final step (**iv**) MRI biomarker maps of oxygen metabolism including oxygen extraction fraction (OEF), cerebral metabolic rate of oxygen (CMRO_2_) [12], and the tissue oxygen tension (PO_2_) [13, 14] were calculated using the following equations:

$$\text{OEF}\text{ }\text{=}\text{ }\frac{\text{R}_{\text{2}}^{\text{*}}\text{-}\text{R}_{\text{2}}}{\frac{\text{4}}{\text{3}}\text{·π·γ·Δχ·Hct·}\text{B}_{\text{0}}\text{ ∙CBV}}$$

with γ (2.67502·10^8^ rad/s/T) is the nuclear gyromagnetic ratio; Δχ = 0.264·10^-6^ is the difference between the magnetic susceptibilities of fully oxygenated and fully deoxygenated haemoglobin; Hct = 0.42·0.85 is the microvascular hematocrit fraction, whereby the factor 0.85 stands for a correction factor of systemic Hct for small vessels;

$$\text{CMRO}_{\text{2}}\text{ }\text{=}\text{ }\text{OEF∙CBF∙}\text{C}_{\text{a}}$$

where C_a_=8.68 mmol/ml is the arterial blood oxygen content [15]; and

$$\text{PO}_{\text{2}}\text{ }\text{=}{\text{ }\text{P}}_{\text{50}}\sqrt[\text{h}]{\left( \frac{\text{2}}{\text{OEF}}\text{-1} \right)} \text{-}\text{ }\frac{\text{CMRO}_{\text{2}}}{\text{L}}$$

where P_50_ is the hemoglobin half-saturation tension of oxygen (27 mmHg), h is the Hill coefficient of oxygen binding to hemoglobin (2.7), and L (4.4 mmol/Hg per minute) is the tissue oxygen conductivity as defined by Vafaee and Gjedde [16].


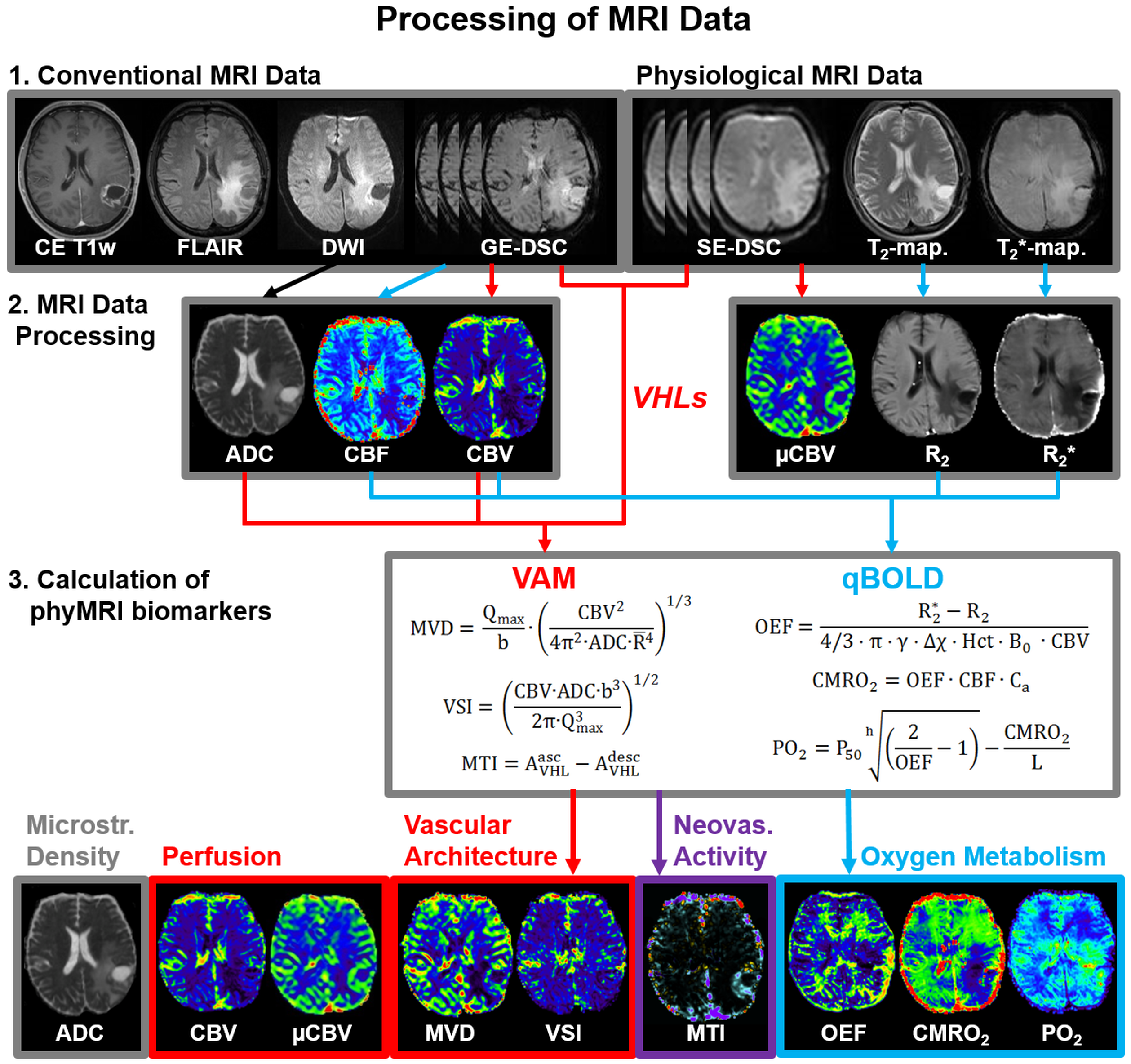


**Supplementary Fig. 1** The pipeline for MRI data processing and calculation of MRI biomarker maps.

**Supplementary Results**

**Supplementary Table 1.** Imaging biomarker values of cMRI and phyMRI for the subgroups of patients with an IDH-mutant astrocytoma WHO grade 3.

|  | **No recurrence** | | | **Recurrence** | | | **cNB** |
| --- | --- | --- | --- | --- | --- | --- | --- |
|  | **Both TN** | **FP phyMRI** | **FP cMRI** | **Simultan. TP** | **early TP phyMRI** | |  |
|  |  |  |  |  | **early FU (FN cMRI)** | **subsequ. FU** |  |
| **ADC**  [mm^2^/s] | 1.18 ± 0.28  0.73 – 1.67 | n/a | 2.87  n/a | 1.30 ± 0.39  0.69 – 1.82 | 1.48 ± 0.35  1.04 – 1.85 | 1.26 ± 0.23  1.02 – 1.68 | 0.82 ± 0.10  0.69 – 1.04 |
| **CBV**  [%] | 4.8 ± 1.7  2.3 – 8.4 | n/a | 2.0  n/a | 19.9 ± 10.7  7.3 – 40.0 | 7.2 ± 2.1  3.7 – 10.6 | 14.5 ± 4.6  7.4 – 21.0 | 6.1 ± 1.6  3.0 – 8.8 |
| **µCBV**  [%] | 2.5 ± 0.7  1.3 – 3.3 | n/a | 1.4  n/a | 6.9 ± 1.4  3.9 – 9.1 | 3.9 ± 1.6  1.5 – 5.7 | 7.0 ± 2.1  3.2 – 9.5 | 3.3 ± 0.7  1.9 – 4.7 |
| **MVD**  [mm^-2^] | 245 ± 38  173 – 308 | n/a | 82  n/a | 409 ± 174  221 – 759 | 182 ± 67  80 – 308 | 348 ± 128  190 – 577 | 217 ± 57  108 – 311 |
| **VSI**  [µm] | 53 ± 11  38 – 75 | n/a | 21  n/a | 77 ± 22  41 – 113 | 64 ± 12  39 – 75 | 61 ± 22  33 – 100 | 44 ± 16  18 – 75 |
| **MTI**  [s^-5/2^] | -0.5 ± 1.0  -2.0 – 1.1 | n/a | 0.1  n/a | -25 ± 20  -67 – -9 | -5.3 ± 7.2  -17.7 – -0.4 | -28 ± 12  -42 – -5.4 | 0.0 ± 1.2  -2.3 – 2.7 |
| **OEF**  [%] | 32 ± 6  24 – 45 | n/a | 81  n/a | 23 ± 19  5 – 69 | 38 ± 14  24 – 63 | 27 ± 14  15 – 52 | 35 ± 9  22 – 59 |
| **CMRO_2_**  [µM/100g∙min] | 83 ± 23  46 – 120 | n/a | 68  n/a | 109 ± 76  32 – 305 | 142 ± 50  74 – 197 | 114 ± 53  53 – 194 | 99 ± 24  55 – 149 |
| **PO_2_**  [mmHg] | 41 ± 9  26 – 57 | n/a | 20  n/a | 55 ± 24  21 – 88 | 29 ± 15  5 – 49 | 53 ± 20  24 – 79 | 33 ± 8  22 – 54 |
| **n** | 14 | 0 | 1 | 12 | 8 | | 35 |

Abbreviations: CBV = cerebral blood volume in macrovasculature; µCBV = CBV in microvasculature; MVD = microvessel density; VSI = vessel size index; MTI = microvessel type indicator; n = patient number in the subgroup; both TN = true negative recurrence detection in cMRI and VAM; FP phyMRI = false positive recurrence detection in physiological MRI; FP cMRI = false positive recurrence detection in cMRI; Simultan. TP = simultaneous and correct WHO grade 3 glioma recurrence detection by both cMRI and phyMRI data; early TP phyMRI = early true positive recurrence detection in phyMRI; early FU = the early follow-up in the “early TP phyMRI” subgroup with a FN finding in the cMRI data; subsequ. FU = the subsequent follow-up in the “early TP phyMRI” subgroup with a delayed TP finding in the cMRI data; cNB = contralateral normal brain; n/a = not available.

**Supplementary Table 2.** Imaging biomarker values of cMRI and phyMRI for the subgroups of patients with an IDH-mutant oligodendroglioma WHO grade 3.

|  | **No recurrence** | | | **Recurrence** | | | **cNB** |
| --- | --- | --- | --- | --- | --- | --- | --- |
|  | **Both TN** | **FP phyMRI** | **FP cMRI** | **Simultan. TP** | **early TP phyMRI** | |  |
|  |  |  |  |  | **early FU (FN cMRI)** | **subsequ. FU** |  |
| **ADC**  [mm^2^/s] | 1.38 ± 0.35  0.91 – 1.77 | 1.07 ± 0.40  0.56 – 1.50 | 1.78 ± 0.22  1.62 – 1.93 | 1.48 ± 0.42  0.73 – 1.89 | 1.40 ± 0.34  0.90 – 1.92 | 1.24 ± 0.29  0.92 – 1.79 | 0.85 ± 0.11  0.70 – 1.10 |
| **CBV**  [%] | 4.6 ± 2.6  2.0 – 8.9 | 10.6 ± 5.8  6.3 – 19.0 | 6.5 ± 4.9  3.0 – 10.0 | 20.5 ± 8.3  14.0 – 36.0 | 5.6 ± 1.2  4.2 – 7.0 | 19.5 ± 5.4  11.6 – 24.7 | 6.5 ± 1.7  3.1 – 8.8 |
| **µCBV**  [%] | 2.2 ± 0.7  1.2 – 3.2 | 5.5 ± 1.8  3.2 – 7.3 | 2.0 ± 0.8  1.4 – 2.5 | 8.9 ± 3.0  5.7 – 13.7 | 3.2 ± 0.8  1.8 – 4.1 | 7.9 ± 2.0  4.7 – 10.2 | 3.2 ± 0.8  1.6 – 4.2 |
| **MVD**  [mm^-2^] | 208 ± 36  147 – 258 | 283 ± 111  122 – 368 | 120 ± 14  110 – 130 | 443 ± 143  296 – 683 | 254 ± 107  86 – 349 | 414 ± 156  216 – 561 | 221 ± 58  130 – 317 |
| **VSI**  [µm] | 54 ± 11  37 – 64 | 67 ± 36  23 – 109 | 89 ± 76  35 – 143 | 79 ± 35  47 – 132 | 58 ± 18  31 – 72 | 69 ± 19  42 – 96 | 46 ± 13  24 – 76 |
| **MTI**  [s^-5/2^] | -1.1 ± 1.2  -2.9 – 0.1 | -6.9 ± 4.2  -10.7 – 1.9 | -0.7 ± 0.8  -1.2 – -0.1 | -37 ± 21  -72 – -18 | -3.7 ± 4.5  -12.6 – -0.6 | -33 ± 10  -48 – -19 | 0.0 ± 1.3  -2.3 – 2.2 |
| **OEF**  [%] | 31 ± 5  25 – 36 | 44 ± 16  28 – 64 | 41 ± 17  29 – 53 | 18 ± 6  9 – 26 | 38 ± 15  28 – 69 | 22 ± 8  13 – 34 | 38 ± 9  23 – 49 |
| **CMRO_2_**  [µM/100g∙min] | 72 ± 28  42 – 111 | 162 ± 62  74 – 211 | 100 ± 62  56 – 144 | 139 ± 99  55 – 342 | 129 ± 64  46 – 197 | 134 ± 52  58 – 190 | 99 ± 31  59 – 153 |
| **PO_2_**  [mmHg] | 41 ± 10  24 – 53 | 26 ± 10  18 – 41 | 41 ± 13  32 – 50 | 55 ± 20  33 – 81 | 31 ± 17  7 – 56 | 50 ± 16  31 – 76 | 37 ± 8  23 – 50 |
| **n** | 6 | 3 | 2 | 7 | 7 | | 25 |

Abbreviations: CBV = cerebral blood volume in macrovasculature; µCBV = CBV in microvasculature; MVD = microvessel density; VSI = vessel size index; MTI = microvessel type indicator; n = patient number in the subgroup; both TN = true negative recurrence detection in cMRI and VAM; FP phyMRI = false positive recurrence detection in physiological MRI; FP cMRI = false positive recurrence detection in cMRI; Simultan. TP = simultaneous and correct WHO grade 3 glioma recurrence detection by both cMRI and phyMRI data; early TP phyMRI = early true positive recurrence detection in phyMRI; early FU = the early follow-up in the “early TP phyMRI” subgroup with a FN finding in the cMRI data; subsequ. FU = the subsequent follow-up in the “early TP phyMRI” subgroup with a delayed TP finding in the cMRI data; cNB = contralateral normal brain.

**Diagnostic performance of MRI biomarkers for recurrence detection of IDH-mutant astrocytoma WHO grade 3 and oligodendroglioma WHO grade 3**

Sensitivity, specificity, accuracy, and precision of cMRI for early recurrence detection of astrocytoma WHO grade 3 were as follows: 0.600, 0.933, 0.743, and 0.923. These values increased for delayed detection with cMRI (considering the subsequent follow-up in the early TP phyMRI subgroup) to 1.0, 0.933, 0.971, and 0.952, respectively. There was neither FP nor FN findings for both early and delayed detection of astrocytoma WHO grade 3 recurrence with phyMRI. Therefore, all parameters for diagnostic performance were 1.0.

For early recurrence detection of IDH-mutant oligodendroglioma WHO grade 3, the parameters for cMRI were as follows: sensitivity = 0.500, specificity = 0.818, accuracy = 0.640, and precision = 0.778; and for phyMRI: sensitivity = 1.0, specificity = 0.727, accuracy = 0.880, and precision = 0.824, respectively. Interestingly, for delayed recurrence detection of oligodendroglioma WHO grade 3, i.e. when considering the subsequent follow-up in the early TP phyMRI subgroup, cMRI showed superior diagnostic performance compared to phyMRI because the parameters for cMRI as follows: sensitivity = 1.0, specificity = 0.818, accuracy = 0.920, and precision = 0.875. The values for phyMRI remained the same.

ROC curve analysis for early recurrence detection of astrocytoma WHO grade 3 revealed highest diagnostic performance for the MRI biomarker representing neovascularization activity MTI (AUC = 0.881) followed by three biomarkers associated with tumor vasculature and perfusion with rather similar diagnostic performance: VSI (0.789), µCBV (0.767), and CBV (0.756). The other parameters showed inferior diagnostic performance for early detection of astrocytoma WHO grade 3 recurrence: ADC (0.696), MVD (0.689), CMRO_2_ (0.678), OEF (0.630), and PO_2_ (0.591). This diagnostic advantage of the biomarkers for neovascularization (MTI: AUC = 1.0), perfusion (CBV: 0.993; µCBV: 0.984), and tumor vasculature (MVD: 0.832; VSI: 0.758) persisted for delayed as well as simultaneous recurrence detection. The biomarkers for oxygen metabolism (OEF: 0.726; PO_2_: 0.704; and CMRO_2_: 0.556) and microstructural density (ADC: 0.502) showed inferior diagnostic performance. **Supplementary Figure 2A** visualizes the changes in the AUC values for recurrence detection of astrocytoma WHO grade 3, *IDH-mutant*.

ROC curve analysis for early recurrence detection of oligodendroglioma WHO grade 3 revealed a similar pattern but in general lower AUC values compared to astrocytoma WHO grade 3: MTI (AUC = 0.773), OEF (0.678), MVD (0.655), µCBV (0.621), CMRO_2_ (0.577), ADC (0.557), VSI (0.527), CBV (0.519), and PO_2_ (0.504). For delayed / simultaneous recurrence detection of oligodendroglioma WHO grade 3, the AUC values were as follows: MTI (0.773), OEF (0.678), MVD (0.655), µCBV (0.621), CMRO_2_ (0.577), ADC (0.557), VSI (0.527), CBV (0.519), and PO_2_ (0.504). Interestingly, OEF showed the second highest AUC values for both early and delayed oligodendroglioma recurrence detection. The changes in the AUC values for recurrence detection of IDH-mutant oligodendroglioma WHO grade 3 are visualized in **Supplementary Figure 2B**.


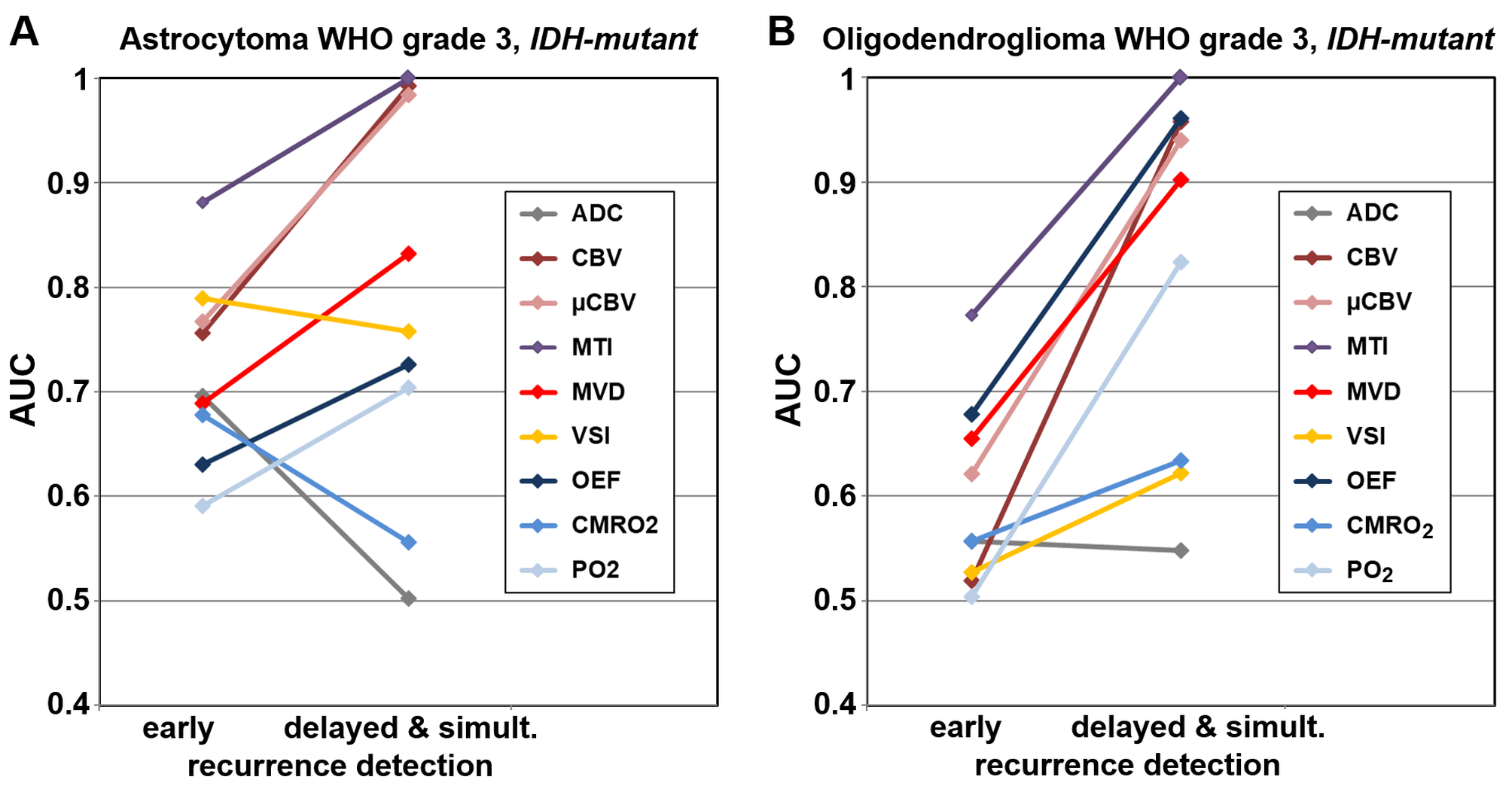


**Supplementary Fig. 2** Changes in the area under the receiver operating characteristic (ROC) curve (AUC) between early recurrence detection and delayed & simultaneous detection of recurrence of **A:** IDH-mutant astrocytoma WHO grade 3, and **B:** IDH-mutant oligodendroglioma WHO grade 3.

**REFERENCES FOR SUPPLEMENTARY MATERIALS**

1. Boxerman JL, Prah DE, Paulson ES, et al (2012) The role of preload and leakage correction in gadolinium-based cerebral blood volume estimation determined by comparison with MION as a criterion standard. Am J Neuroradiol 33:1081–1087

2. Boxerman JL, Schmainda KM, Weisskoff RM (2006) Relative cerebral blood volume maps corrected for contrast agent extravasation significantly correlate with glioma tumor grade, whereas uncorrected maps do not. Am J Neuroradiol 27:859–867

3. Stadlbauer A, Zimmermann M, Heinz G, et al (2017) Magnetic resonance imaging biomarkers for clinical routine assessment of microvascular architecture in glioma. J Cereb blood flow Metab 37:632–643

4. Ducreux D, Buvat I, Meder JF, et al (2006) Perfusion-weighted MR imaging studies in brain hypervascular diseases: comparison of arterial input function extractions for perfusion measurement. AJNR Am J Neuroradiol 27:1059–1069

5. Xu C, Kiselev VG, Möller HE, Fiebach JB (2013) Dynamic hysteresis between gradient echo and spin echo attenuations in dynamic susceptibility contrast imaging. Magn Reson Med 69:981–991

6. Stadlbauer A, Zimmermann M, Oberndorfer S, et al (2017) Vascular Hysteresis Loops and Vascular Architecture Mapping in Patients with Glioblastoma treated with Antiangiogenic Therapy. Sci Rep 7:1–12

7. Jensen JH, Lu H, Inglese M (2006) Microvessel density estimation in the human brain by means of dynamic contrast-enhanced echo-planar imaging. Magn Reson Med 56:1145–1150

8. Bjornerud A, Emblem KE (2010) A fully automated method for quantitative cerebral hemodynamic analysis using DSC-MRI. J Cereb Blood Flow Metab 30:1066–1078

9. Preibisch C, Volz S, Anti S, Deichmann R (2008) Exponential excitation pulses for improved water content mapping in the presence of background gradients. Magn Reson Med 60:908–916

10. Prasloski T, Mädler B, Xiang QS, et al (2012) Applications of stimulated echo correction to multicomponent T2 analysis. Magn Reson Med 67:1803–1814

11. Smith AM, Grandin CB, Duprez T, et al (2000) Whole Brain Quantitative CBF, CBV, and MTT Measurements Using MRI Bolus Tracking: Implementation and Application to Data Acquired From Hyperacute Stroke Patients. J Magn Reson Imaging 12:400–410

12. Christen T, Schmiedeskamp H, Straka M, et al (2012) Measuring brain oxygenation in humans using a multiparametric quantitative blood oxygenation level dependent MRI approach. Magn Reson Med 68:905–911

13. Gjedde A (2002) Cerebral blood flow change in arterial hypoxemia is consistent with negligible oxygen tension in brain mitochondria. Neuroimage 17:1876–1881

14. Vafaee MS, Vang K, Bergersen LH, Gjedde A (2012) Oxygen consumption and blood flow coupling in human motor cortex during intense finger tapping: implication for a role of lactate. J Cereb Blood Flow Metab 32:1859–68

15. Kennan RP, Zhong J, Gore JC (1994) Intravascular susceptibility contrast mechanisms in tissues. Magn Reson Med 31:9–21

16. Vafaee MS, Gjedde A (2000) Model of blood-brain transfer of oxygen explains nonlinear flow-metabolism coupling during stimulation of visual cortex. J Cereb blood flow Metab 20:747–754
